# Characterization of the complete mitochondrial genome of biocontrol yeast *Sporobolomyces* sp. (Sporidiobolales: Sporidiobolaceae) with phylogenetic analysis

**DOI:** 10.1080/23802359.2020.1797581

**Published:** 2020-07-28

**Authors:** Jingwei Huang, Huijuan Qu, Xueshan Shen

**Affiliations:** a College of Medicine (School of Nursing), Chengdu University, Chengdu, P.R. China; b Key Laboratory of Coarse Cereal Processing, Ministry of Agriculture and Rural Affairs, Chengdu University, Chengdu, P.R. China; c Sichuan Academy of Agricultural Sciences, Chengdu, P.R. China

**Keywords:** Yeast, mitochondrial genome, phylogenetic analysis, evolution

## Abstract

In this study, we obtained the complete mitochondrial genome of *Sporobolomyces* sp. using next-generation sequencing. The complete mitochondrial genome of *Sporobolomyces* sp. contained 15 protein-coding genes (PCG), two ribosomal RNA (rRNA) genes, and 25 transfer RNA (tRNA) genes. The total length of the *Sporobolomyces* sp. mitochondrial genome is 26,430 bp, and the GC content of the mitochondrial genome is 39.32%. Phylogenetic analysis based on combined mitochondrial gene dataset indicated that the mitochondrial genome of *Sporobolomyces* sp. exhibited a close relationship with that of *Rhodotorula mucilaginosa*.

The genus *Sporobolomyces* is a group of ballistoconidium-forming yeast. Dozens of species have been described in this genus (Satoh and Makimura 2008; Lorenzini et al. 2019). *Sporobolomyces* species are distributed in various habitats, such as on plant phyllodes, on the surface area of soils, and also in the fermentation system (Barahona et al. 2016; Arrigoni et al. 2018; Li, Yuan, et al. 2020). Limited morphological characteristics make it difficult to identify *Sporobolomyces* species accurately only by morphology (Hamamoto and Nakase 2000; Barahona et al. 2016; Lorenzini et al. 2019). Mitochondrial genome has been widely used in the phylogenetic analysis of species (Wang et al. 2020; Li, He, et al. 2020). However, up to now, no mitochondrial genome from the genus *Sporobolomyces* has been reported. The mitochondrial genome of *Sporobolomyces* sp. will promote the understanding of the phylogeny, evolution, and taxonomy of this important yeast.

The specimen (*Sporobolomyces* sp.) was collected from Jilin, China (129.32 E; 42.53 N). The specimen was stored in the Culture Collection Center of Chengdu University (No. Spo_ns1). The complete mitochondrial genome of *Sporobolomyces* sp. was sequenced and assembled according to previously described methods (Li, Liao, et al. 2018; Li, Xiang, et al. 2019; Wang et al. 2020). Briefly, the total genomic DNA of *Sporobolomyces* sp. was extracted using a Fungal DNA Kit D3390-00 (Omega Bio-Tek, Norcross, GA, USA). The extracted genomic DNA was purified using a Gel Extraction Kit (Omega Bio-Tek, Norcross, GA, USA). The purified DNA was stored in Chengdu University (No. DNA_ Spo_ns1). We constructed sequencing libraries with the purified genomic DNA using a NEBNext^®^ Ultra™ II DNA Library Prep Kit (NEB, Beijing, China). Whole genomic sequencing (WGS) of *Sporobolomyces* sp. was conducted using the Illumina HiSeq 2500 Platform (Illumina, SanDiego, CA). We *de novo* assembled the mitochondrial genome of *Sporobolomyces* sp. using SPAdes 3.9.0 (Bankevich et al. 2012; Li, Ren, et al. 2020). The complete mitochondrial genome of *Sporobolomyces* sp. was annotated according to previously described methods (Li, Chen, et al. 2018; Li, Wang, et al. 2018).

We found the complete mitochondrial genome of *Sporobolomyces* sp. is 26,430 bp in length, with the base composition as follows: A (30.82%), T (29.84%), G (20.35%), and C (18.97%). The complete mitochondrial genome of *Sporobolomyces* sp. contains 15 protein-coding genes, two ribosomal RNA genes (*rns* and *rnl*), and 25 transfer RNA (tRNA) genes. To investigate the phylogenetic positions of *Sporobolomyces* sp., we constructed a phylogenetic tree for 18 Basidiomycota species. *Rhizopogon salebrosus* from the Boletales order was set as the outgroup (Li, Ren, et al. 2019). The phylogenetic tree was constructed using the Bayesian analysis (BI) method based on the combined 14 core protein-coding genes according to previously described methods (Li, Wang, Jin, Chen, Xiong, Li, Liu, et al. 2019; Li, Wang, Jin, Chen, Xiong, Li, Zhao, et al. 2019; Li, Yang, et al. 2020). As shown in the phylogenetic tree (Figure 1), the mitochondrial genome of *Sporobolomyces* sp. exhibited a close relationship with that of *Rhodotorula mucilaginosa* (Gan et al. 2017).

**Figure 1.**
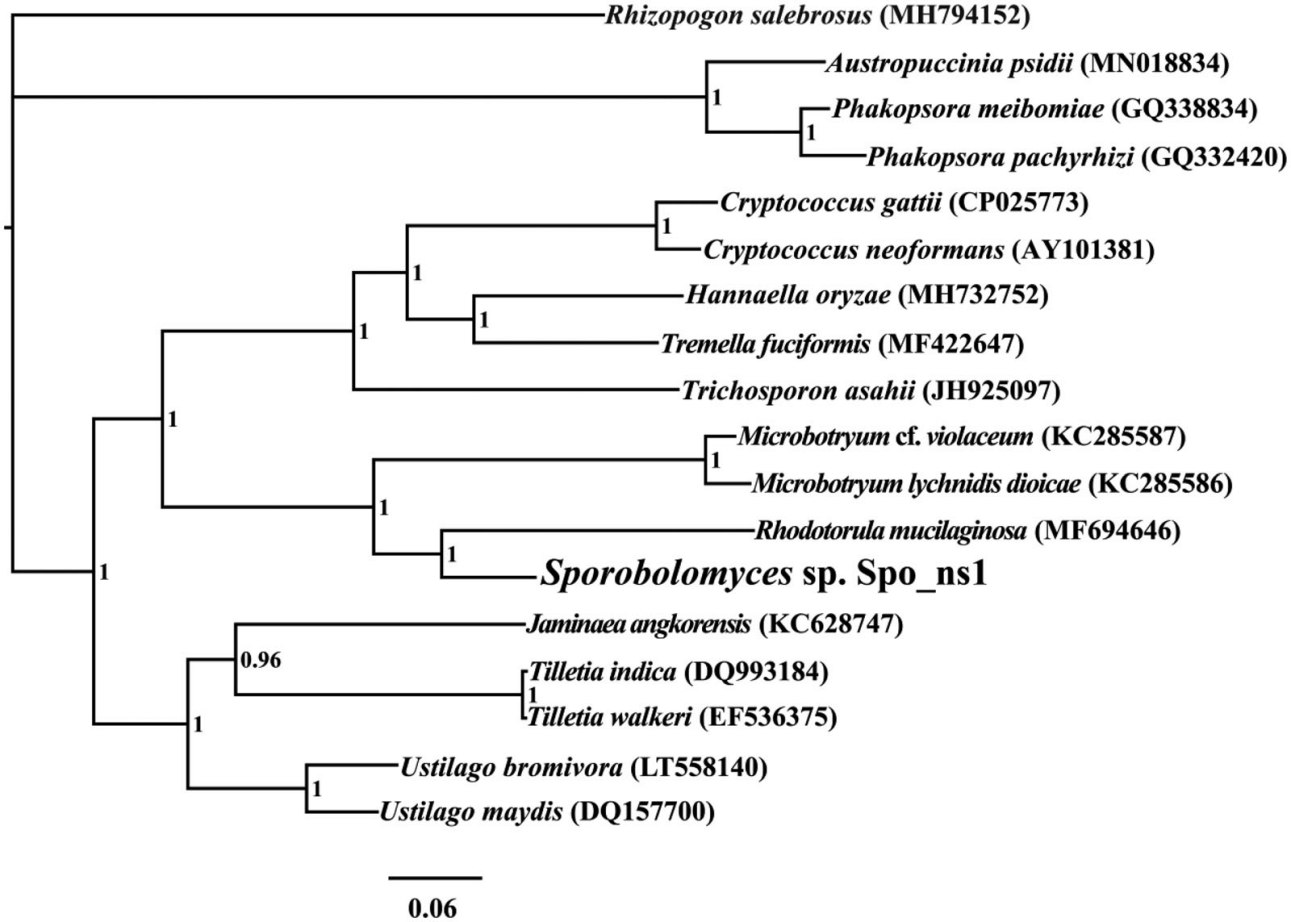
Bayesian phylogenetic analysis of 18 species based on the combined 14 core protein-coding genes. Accession numbers of mitochondrial sequences used in the phylogenetic analysis are listed in brackets after species.

## Data Availability

This mitogenome of *Sporobolomyces* sp. was submitted to GenBank under the accession number of MT663151. (https://www.ncbi.nlm.nih.gov/nuccore/MT663151).

## References

[CIT0001] Arrigoni E, Antonielli L, Pindo M, Pertot I, Perazzolli M. 2018. Tissue age and plant genotype affect the microbiota of apple and pear bark. Microbiol Res. 211:57–68. 29705206 10.1016/j.micres.2018.04.002

[CIT0002] Bankevich A, Nurk S, Antipov D, Gurevich AA, Dvorkin M, Kulikov AS, Lesin VM, Nikolenko SI, Pham S, Prjibelski AD, et al. 2012. SPAdes: a new genome assembly algorithm and its applications to single-cell sequencing. J Comput Biol. 19(5):455–477. 22506599 10.1089/cmb.2012.0021 PMC3342519

[CIT0003] Barahona S, Yuivar Y, Socias G, Alcaino J, Cifuentes V, Baeza M. 2016. Identification and characterization of yeasts isolated from sedimentary rocks of Union Glacier at the Antarctica. Extremophiles. 20(4):479–491. 27215207 10.1007/s00792-016-0838-6

[CIT0004] Gan HM, Thomas BN, Cavanaugh NT, Morales GH, Mayers AN, Savka MA, Hudson AO. 2017. Whole genome sequencing of *Rhodotorula mucilaginosa* isolated from the chewing stick (*Distemonanthus benthamianus*): insights into Rhodotorula phylogeny, mitogenome dynamics and carotenoid biosynthesis. PeerJ. 5:e4030. 29158974 10.7717/peerj.4030 PMC5691792

[CIT0005] Hamamoto M, Nakase T. 2000. Phylogenetic analysis of the ballistoconidium-forming yeast genus *Sporobolomyces* based on 18S rDNA sequences. Int J Syst Evol Microbiol. 50 Pt 3:1373–1380. 10.1099/00207713-50-3-1373 10843083

[CIT0006] Li Q, Chen C, Xiong C, Jin X, Chen Z, Huang W. 2018. Comparative mitogenomics reveals large-scale gene rearrangements in the mitochondrial genome of two *Pleurotus* species. Appl Microbiol Biotechnol. 102(14):6143–6153. 29799088 10.1007/s00253-018-9082-6

[CIT0007] Li Q, He X, Ren Y, Xiong C, Jin X, Peng L, Huang W. 2020. Comparative mitogenome analysis reveals mitochondrial genome differentiation in ectomycorrhizal and asymbiotic *Amanita* species. Front Microbiol. 11:1382. 32636830 10.3389/fmicb.2020.01382 PMC7318869

[CIT0008] Li Q, Liao M, Yang M, Xiong C, Jin X, Chen Z, Huang W. 2018. Characterization of the mitochondrial genomes of three species in the ectomycorrhizal genus *Cantharellus* and phylogeny of Agaricomycetes. Int J Biol Macromol. 118:756–769. 29959010 10.1016/j.ijbiomac.2018.06.129

[CIT0009] Li Q, Ren Y, Shi X, Peng L, Zhao J, Song Y, Zhao G. 2019. Comparative mitochondrial genome analysis of two ectomycorrhizal fungi (*Rhizopogon*) reveals dynamic changes of intron and phylogenetic relationships of the subphylum Agaricomycotina. IJMS. 20(20):5167. 10.3390/ijms20205167 PMC6829451 31635252

[CIT0010] Li Q, Ren Y, Xiang D, Shi X, Zhao J, Peng L, Zhao G. 2020. Comparative mitogenome analysis of two ectomycorrhizal fungi (*Paxillus*) reveals gene rearrangement, intron dynamics, and phylogeny of basidiomycetes. IMA Fungus. 11(1):12. 32670777 10.1186/s43008-020-00038-8 PMC7333402

[CIT0011] Li Q, Wang Q, Chen C, Jin X, Chen Z, Xiong C, Li P, Zhao J, Huang W. 2018. Characterization and comparative mitogenomic analysis of six newly sequenced mitochondrial genomes from ectomycorrhizal fungi (*Russula*) and phylogenetic analysis of the Agaricomycetes. Int J Biol Macromol . 119:792–802. 30076929 10.1016/j.ijbiomac.2018.07.197

[CIT0012] Li Q, Wang Q, Jin X, Chen Z, Xiong C, Li P, Liu Q, Huang W. 2019. Characterization and comparative analysis of six complete mitochondrial genomes from ectomycorrhizal fungi of the *Lactarius* genus and phylogenetic analysis of the Agaricomycetes. Int J Biol Macromol. 121:249–260. 30308282 10.1016/j.ijbiomac.2018.10.029

[CIT0013] Li Q, Wang Q, Jin X, Chen Z, Xiong C, Li P, Zhao J, Huang W. 2019. Characterization and comparison of the mitochondrial genomes from two *Lyophyllum* fungal species and insights into phylogeny of Agaricomycetes. Int J Biol Macromol. 121:364–372. 30315880 10.1016/j.ijbiomac.2018.10.037

[CIT0014] Li Q, Xiang D, Wan Y, Wu Q, Wu X, Ma C, Song Y, Zhao G, Huang W. 2019. The complete mitochondrial genomes of five important medicinal *Ganoderma* species: features, evolution, and phylogeny. Int J Biol Macromol. 139:397–408. 31381907 10.1016/j.ijbiomac.2019.08.003

[CIT0015] Li Q, Yang L, Xiang D, Wan Y, Wu Q, Huang W, Zhao G. 2020. The complete mitochondrial genomes of two model ectomycorrhizal fungi (*Laccaria*): features, intron dynamics and phylogenetic implications. Int J Biol Macromol. 145:974–984. 31669472 10.1016/j.ijbiomac.2019.09.188

[CIT0016] Li AH, Yuan FX, Groenewald M, Bensch K, Yurkov AM, Li K, Han PJ, Guo LD, Aime MC, Sampaio JP, Jindamorakot S, et al. 2020. Diversity and phylogeny of basidiomycetous yeasts from plant leaves and soil: proposal of two new orders, three new families, eight new genera and one hundred and seven new species. Stud Mycol. 96:17–140. 32206137 10.1016/j.simyco.2020.01.002 PMC7082220

[CIT0017] Lorenzini M, Zapparoli G, Azzolini M, Carvalho C, Sampaio JP. 2019. *Sporobolomyces agrorum* sp. nov. and *Sporobolomyces sucorum* sp. nov., two novel basidiomycetous yeast species isolated from grape and apple must in Italy. Int J Syst Evol Microbiol. 69(11):3385–3391. 31368884 10.1099/ijsem.0.003626

[CIT0018] Satoh K, Makimura K. 2008. *Sporobolomyces koalae* sp. nov., a basidiomycetous yeast isolated from nasal smears of Queensland koalas kept in a Japanese zoological park. Int J Syst Evol Microbiol. 58(Pt 12):2983–2986. 19060093 10.1099/ijs.0.2008/000307-0

[CIT0019] Wang X, Song A, Wang F, Chen M, Li X, Li Q, Liu N. 2020. The 206 kbp mitochondrial genome of *Phanerochaete carnosa* reveals dynamics of introns, accumulation of repeat sequences and plasmid-derived genes. Int J Biol Macromol. 162:209–219. 32562727 10.1016/j.ijbiomac.2020.06.142

